# Short-Term Longitudinal Biometric Identification of Cattle Using Planum Nasolabiale Images and a Two-Stage Deep Learning Framework

**DOI:** 10.3390/vetsci13090924

**Published:** 2026-09-08

**Authors:** Selahattin Ozan Yardımcı, İmdat Orhan, Fatih İsmail Bahar

**Affiliations:** 1 Department of Anatomy, Faculty of Veterinary Medicine, Erciyes University, 38280 Talas, Kayseri, Türkiye; znyrdmc@gmail.com; 2 Faculty of Veterinary Medicine, Erciyes University, 38280 Talas, Kayseri, Türkiye; 1080111722@erciyes.edu.tr

**Keywords:** cattle identification, planum nasolabiale, muzzle pattern, biometric identification, longitudinal validation, deep learning, YOLOv12, EfficientNet, precision livestock farming

## Abstract

Individual cattle identification is essential for traceability and precision livestock management, but conventional identifiers may be lost or damaged. This study evaluated a non-invasive biometric framework based on PN images. A two-stage pipeline combined a YOLOv12n detector for muzzle localization with an ImageNet-pretrained EfficientNetB0 classifier for individual identification. Forty-eight cattle (32 adult cows and 16 calves) were photographed on 31 January 2025 (T0) and again on 11 June 2025 (T1), a 131-day interval. Four images per animal were obtained at each session, producing 384 longitudinal images. The detector-development dataset comprised 565 original images from cattle sampled independently at a slaughterhouse and 210 public Zenodo images, with no animal- or image-level overlap with the longitudinal dataset. The 775 detector images were randomly partitioned at image level into training (*n* = 543), validation (*n* = 116), and test (*n* = 116) subsets before augmentation; online augmentation was applied only to the training subset. Muzzle detection achieved precision of 0.890, recall of 0.978, and mAP@0.5 of 0.982. The muzzle was localized in all validation and test images, with a reported mean intersection over union of 0.96. All T1 images were excluded from model development and used as an independent closed-set temporal test, yielding 96.3% overall re-identification accuracy. End-to-end inference required 8.3 ms per image on an NVIDIA L4 GPU. The results support short-term retention of discriminative PN characteristics, including in a cohort containing growing calves, and indicate that PN imaging may serve as a complementary cattle-identification tool.

## Simple Summary

Reliable individual identification is fundamental to cattle traceability, herd management, disease control, and animal welfare. Conventional identifiers such as ear tags remain essential but may be lost, damaged, or difficult to read. The planum nasolabiale (PN), the hairless surface between the nostrils and upper lip, has an individually distinctive pattern that can be photographed without touching the animal. This study trained a two-stage computer-vision system using baseline PN images and tested whether the same 48 cattle could be re-identified in a fully separate imaging session 131 days later. The cohort included 32 adult cows and 16 calves, allowing short-term evaluation during a period in which growth-related change was expected to be more pronounced in the calves. The system correctly classified 185 of 192 follow-up images, corresponding to 96.35% overall longitudinal re-identification accuracy (95% CI: 92.67–98.22%). The system achieved 96.3% overall re-identification accuracy on the 192 follow-up images. These findings support PN imaging as a promising non-invasive complement to established cattle identification methods, while longer follow-up, multi-farm validation, age-stratified analysis, and open-set testing remain necessary before routine deployment.

## 1. Introduction

Accurate individual identification underpins livestock traceability, disease surveillance, breeding records, food-chain assurance, and precision management. Ear tags, tattoos, branding, and radio-frequency identification are widely used, but physical identifiers can be lost, damaged, misread, or intentionally removed, and some procedures require restraint or tissue injury [1,2]. Image-based biometric methods are therefore of interest as complementary tools because they use anatomical characteristics that are intrinsic to the animal.

In cattle, the PN is the hairless, normally moist integumentary region extending between the nostrils and the upper lip. Petersen first proposed bovine nose prints for individual identification [3], and Hirsch et al. subsequently developed a systematic classification of bovine nose-print patterns [4]. Studies in cattle, carabaos, and Holstein–Friesian crosses further described the practical use and anatomical organization of the muzzle surface [5,6]. The PN contains papillae, ridges, grooves, beads, islands, and polygonal fields whose combination varies among individuals. These structures form the anatomical basis for a biometric representation, although their apparent appearance can change with illumination, viewpoint, moisture, contamination, and growth.

Early computational work moved from manual print comparison toward digital feature analysis. Niu and Sasaki evaluated local image features for cattle muzzle recognition [7]. Barry et al. demonstrated a digital biometric approach based on muzzle patterns [8], whereas Kimura and Watanabe used flexible graph matching to accommodate deformation associated with growth [9]. Together, these studies established that useful identity information can be extracted from the PN while also emphasizing the need to account for geometric and temporal variation.

Deep learning has expanded the scale and automation of cattle biometrics. Shojaeipour et al. combined YOLOv3 muzzle localization with ResNet50 identification and few-shot transfer learning in 300 mixed-breed cattle [10]. Li et al. benchmarked 59 classification architectures using 4923 muzzle images from 268 beef cattle and reported a maximum accuracy of 98.7% and a fastest processing time of 28.3 ms per image [11]. Multi-feature fusion of the face, muzzle, and ear tag has been proposed for complex feeding and drinking scenes in which a single feature may be obscured [12]. Open-set metric-learning systems have also been developed to reject previously unseen cattle rather than forcing every image into a registered identity [13]. However, most published benchmarks are cross-sectional or use random image partitions; a high score under those conditions does not establish that the same individual will be recognized after a genuinely separate acquisition session (Table 1).

The primary aim of this study was to determine whether an artificial intelligence system developed using baseline PN images could re-identify the same cattle in an independent imaging session conducted 131 days later. The secondary aim was to evaluate whether the discriminative PN characteristics of a cohort containing calves remained sufficiently stable for re-identification during this growth period. The study was designed as a closed-set feasibility evaluation: all identities present at T1 were registered at T0. Muzzle and straw detection supported automated PN localization, whereas open-set rejection and lifelong persistence were outside the scope of the present experiment.

## 2. Materials and Methods

### 2.1. Study Design, Animals, and Image Acquisition

The study was conducted at the Erciyes University Agricultural Research and Application Center (ERÜTAM), Kayseri, Türkiye. All cattle were female Holsteins owned, housed, and managed by ERÜTAM; no privately owned animals were included. The study population consisted of 32 adult cows aged 3–6 years and 16 calves aged 3–6 months. Written institutional permission was obtained from the ERÜTAM Directorate before data collection (document no. E-60875766-604.01.01-522355; 6 October 2023).

The longitudinal cohort comprised 48 cattle: 32 adult cows and 16 calves. The same 48 animals were photographed in two mutually independent sessions. Baseline imaging (T0) was performed on 31 January 2025. Four frontal or near-frontal images were obtained from different angles for each animal (192 images). Follow-up imaging (T1) was performed on 11 June 2025, 131 days after T0. Four new images were obtained from each of the same animals (192 images). The age-group composition remained 32 adult cows and 16 calves at both sessions. The longitudinal dataset therefore contained 384 original images (Table 2).

The same operator acquired the T0 and T1 photographs using the same Canon EOS R50 camera. Images were obtained under routine farm conditions. The operator positioned the camera to provide a clear frontal or near-frontal view of the PN while allowing natural variation in head position, viewing angle, illumination, and surface contamination. No treatment, biological sampling, experimental manipulation, additional restraint, or alteration of routine management was performed. The two sessions were kept separate by acquisition date, and no T1 image was used during model development.

A separate detector-development dataset comprising 565 images from independent slaughterhouse cattle and 210 images from the publicly available Muzzle Cow New Dataset on Zenodo was used exclusively for muzzle and straw localization. This dataset contained no animals or images from the 48-cattle longitudinal cohort and is described separately in Section 2.3.

### 2.2. Ethical and Institutional Considerations

Only non-invasive photographs were obtained under routine husbandry conditions. The animals were not subjected to experimental intervention, treatment, biological sampling, additional restraint, or handling beyond normal farm practice. On this basis, ethical review was waived. Institutional authorization for photography was documented by ERÜTAM before image acquisition.

### 2.3. Detector Dataset, Annotation, and Partitioning

The detector-development dataset comprised 775 original images. Of these, 565 were locally acquired from randomly selected cattle at a slaughterhouse, and 210 were obtained from the publicly available Muzzle Cow New Dataset on Zenodo [14]. The public dataset contains 150 training and 60 test images from 30 cattle identities and was used under the Creative Commons Attribution 4.0 license. The 565 slaughterhouse images were obtained from animals completely independent of the 48 ERÜTAM cattle. Consequently, the detector-development and longitudinal identification datasets had no animal- or image-level overlap.

All 775 detector images were pooled and randomly partitioned at image level into training (*n* = 543), validation (*n* = 116), and test (*n* = 116) subsets before augmentation. Images were annotated in LabelMe [15] using bounding boxes. Two object classes were defined: muzzle and straw. Each image contained one muzzle annotation, whereas the number of straw objects varied, allowing the detector to encounter images with no visible straw and images with partial contamination.

### 2.4. YOLOv12n Muzzle and Straw Detection

YOLOv12n was selected because its attention-centric architecture combines object-detection accuracy with low inference latency [16]. Training used 1024 × 1024-pixel inputs, a batch size of 16, 100 epochs, AdamW optimization, and an initial learning rate of 0.002. Online augmentation comprised mosaic augmentation, copy–paste, hue-saturation-value color manipulation, horizontal flipping, and scaling. Augmentation was applied exclusively to the 543-image training subset; the 116 validation and 116 test images remained in their original form. Automatic mixed precision (AMP) was used during training. Loss weights were 7.5 for bounding-box loss, 0.5 for classification loss, and 1.5 for distribution focal loss. Training was performed on an NVIDIA L4 graphics processing unit (GPU).

Detector performance was summarized using precision, recall, mean average precision at an intersection-over-union threshold of 0.5 (mAP@0.5), and mean intersection over union (IoU). Muzzle localization success was also recorded for the complete validation and test subsets.

### 2.5. PN Extraction and Individual Identification

Muzzle regions detected by YOLOv12n were cropped and supplied to the individual-identification model. An EfficientNetB0 network pretrained on ImageNet was used as the classifier [17]. PN crops were resized to 224 × 224 pixels and supplied in the 0–255 range to the model’s internal rescaling operation. The first 100 layers of the pretrained backbone were kept frozen, while the remaining layers and the 48-class classification head were optimized. The head comprised global average pooling, 30% dropout, and a fully connected output layer. L2 regularization (0.0001) and categorical cross-entropy loss were used.

The classifier was optimized with Adam for up to 50 epochs. The learning rate increased from 0.0001 to 0.001 during the first five epochs, remained at 0.001 through epoch 30, and was subsequently reduced to 0.0005 and 0.00025. Early stopping was configured with a patience of 10 epochs. The 192 T0 images constituted the classifier-development and enrollment dataset and were partitioned at image level into 134 training images, 29 validation images, and 29 internal-test images, corresponding approximately to a 70%/15%/15% split. An identity-coverage constraint ensured that at least one image from each of the 48 cattle identities was included in the training subset; therefore, all target classes were represented during classifier training. Because only four T0 images were available per animal, full identity stratification across both the validation and internal-test subsets was not feasible. Online augmentation was applied exclusively to the 134-image training subset and included rotations of up to 15 degrees, zooming from 0.9 to 1.1, horizontal flipping, and brightness adjustment; the 29 validation and 29 internal-test images remained in their original form. All 192 T1 images were kept completely separate from this partition and were evaluated only after model development as the independent longitudinal test. The classifier was evaluated as a single-run model-development experiment. The precise random seed used in the original workflow was not documented in the archived study records; therefore, no retrospective seed value was assigned.

A rule-based straw-masking procedure based on conversion to the CIELAB color space and Otsu thresholding of the L channel [18] was explored during development. Because alternative masking controls were not part of the retained experimental design, the revised manuscript does not treat the mask as a validated causal intervention and does not report comparative masking percentages.

### 2.6. Outcomes, Explainability, and Computational Performance

The primary outcome was image-level closed-set identification accuracy on the 192 T1 images. Accuracy was calculated as the number of correctly classified images divided by the total number of T1 images, and the 95% confidence interval was calculated using the Wilson score method for a binomial proportion. The temporal test contained 128 images from adult cows and 64 images from calves. The retained T1 result was a pooled cohort-level estimate; no formal age-group comparison is claimed. The identification model used a 48-class softmax output layer and was designed for closed-set 1:N identification. All cattle evaluated at T1 had been enrolled at T0. The model did not include an unknown-identity class or a predefined confidence- or distance-based rejection threshold; therefore, open-set rejection and the associated false-acceptance and false-rejection rates were not evaluated. Detector outcomes were evaluated separately on its validation and test subsets. Gradient-weighted class activation mapping (Grad-CAM) was used only for qualitative inspection of classifier attention and was not treated as quantitative evidence [19]. Computational latency was measured on the NVIDIA L4 GPU for the detector, classifier, and complete pipeline. The study was treated as a descriptive feasibility evaluation; no inferential claim is made for exploratory preprocessing procedures.

### 2.7. Explainability, Field Evaluation, and Computational Performance

Gradient-weighted class activation mapping (Grad-CAM) was used to visualize image regions contributing to classifier decisions [19]. The thesis also reported a real-time field evaluation involving the 32 adult cows, with approximately 15 views per animal under varying image conditions. Computational latency was measured on an NVIDIA L4 GPU for the detector, classifier, and complete pipeline.

### 2.8. Use of Generative Artificial Intelligence

OpenAI ChatGPT Work (OpenAI, San Francisco, CA, USA; https://chatgpt.com; accessed 29–31 July 2026) was used during manuscript preparation to assist with Turkish-to-English translation, English-language editing, condensation and organization of thesis-derived text, adaptation of the manuscript to the journal structure, and refinement of tables, figure captions, author statements, and reference formatting. The tool was also used to suggest clearer wording for methodological descriptions and study limitations and to identify potentially relevant literature. It was not used for data collection, image annotation, scientific image generation, model training, generation of model predictions, calculation of performance metrics, or statistical analysis. All AI-generated text and bibliographic suggestions were critically reviewed by the authors and checked against the original thesis records and cited sources. All scientific interpretations and final editorial decisions were made by the authors, who take full responsibility for the content of the manuscript.

## 3. Results

### 3.1. Detector Performance

**Figure 1:**
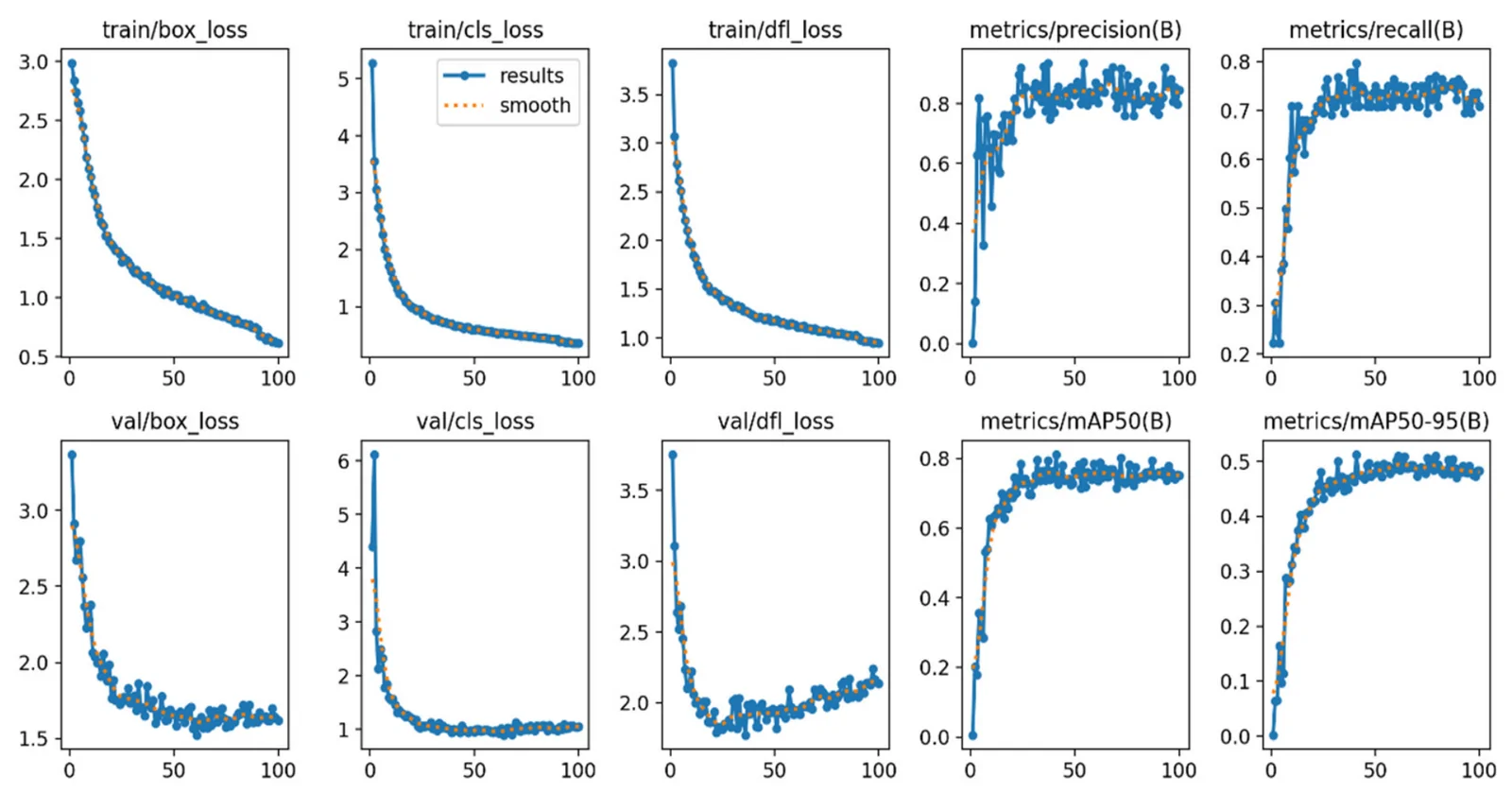
YOLOv12n detector training and validation curves over 100 epochs. The plots show bounding-box, classification, and distribution focal losses together with precision, recall, mAP@0.5, and mAP@0.5:0.95 trajectories.

The best combined mAP@0.5 was 0.785 at epoch 43. At the final epoch, combined precision, recall, and mAP@0.5 were 0.812, 0.764, and 0.754, respectively. Class-specific performance was strongest for the muzzle class, with precision 0.890, recall 0.978, and mAP@0.5 0.982. Straw detection was more difficult, with precision 0.734, recall 0.578, and mAP@0.5 0.588 (Figure 1, Table 3).

**Figure 2:**
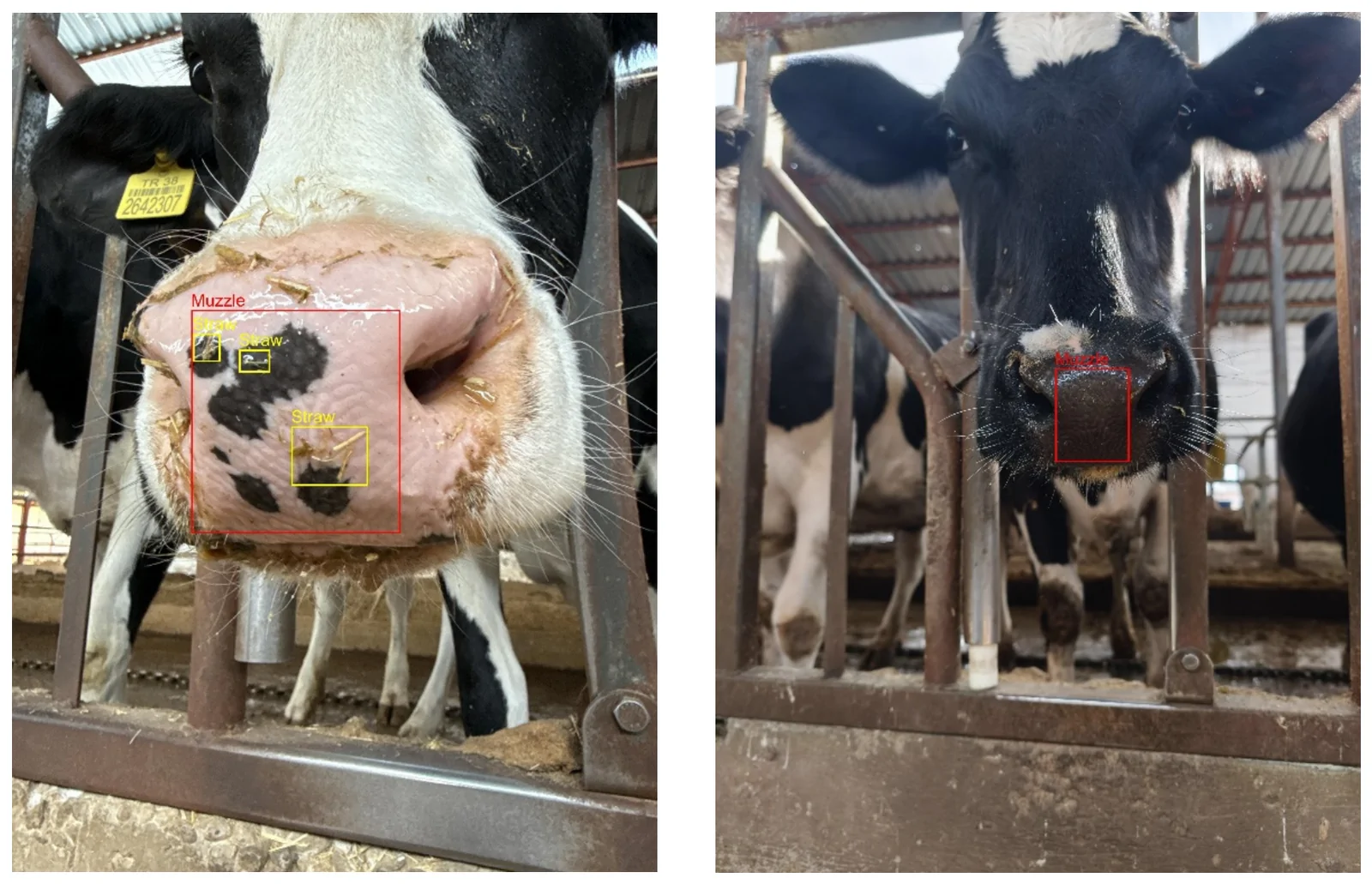
Representative automated localization of the muzzle (red box) and visible straw fragments (yellow boxes) in cattle images.

The detector localized the muzzle in all 116 validation and all 116 test images (232/232 images). The reported mean IoU was 0.96 for muzzle localization and 0.76 for straw localization. The lower straw performance reflected irregular straw shape and occasional confusion with bright, straw-like image regions (Figure 2).

### 3.2. Independent 131-Day Re-Identification

All 192 T1 images acquired on 11 June 2025 were evaluated as a closed-set temporal test. The system correctly classified 185 of 192 images, corresponding to 96.35% overall longitudinal re-identification accuracy (95% CI: 92.67–98.22%; Wilson score method); seven images were incorrectly classified. The evaluation included four new images from every registered animal: 128 images from the 32 adult cows and 64 images from the 16 calves. Because no T1 image was used for model development, the result represents performance across an independent acquisition session rather than a random image split from a single session.

The pooled result indicates that the PN retained sufficient discriminative information for short-term re-identification across 131 days. Because age-stratified prediction summaries were not used as a separate endpoint, the finding should not be interpreted as proof that calf and adult performance were identical or that PN morphology remains unchanged throughout life.

### 3.3. Computational Performance

The YOLOv12n detector required 4.6 ms per 1024 × 1024 image (approximately 217 frames/s), and EfficientNetB0 required 3.2 ms per 224 × 224 PN crop (approximately 312 frames/s). The complete detector-classifier pipeline required 8.3 ms per image, corresponding to approximately 120 frames/s on the NVIDIA L4 GPU (Table 4).

## 4. Discussion

The main finding was that a classifier developed from baseline PN images could re-identify the same 48 cattle in a fully separate imaging session 131 days later, correctly classifying 185 of 192 images (96.35%; 95% CI: 92.67–98.22%). This temporal separation is central to the study. It reduces the risk that performance reflects only near-duplicate photographs captured during a single session and provides a direct short-term test of whether discriminative PN information persists across changes in date, animal position, illumination, surface condition, and growth.

The result is consistent with the anatomical premise established by classical bovine nose-print research. Petersen [3] and Hirsch et al. [4] described individually distinctive pattern organization, whereas subsequent studies documented muzzle-print morphology in cattle and related bovids [5,6,20,21,22,23,24,25,26,27,28]. These works support the existence of stable ridges, grooves, beads, islands, and papillary fields. Nevertheless, a high re-identification rate should be interpreted as retention of discriminative image features, not as proof that every morphological detail remained unchanged. Moisture, skin deformation, illumination, camera angle, contamination, and growth can alter the visible representation even when identity-relevant structure persists.

The inclusion of 16 calves is relevant because proportional growth and soft-tissue change are expected to be greater in young animals than in adult cows. All calves contributed four images at T0 and four new images at T1. The pooled 96.35% result therefore demonstrates that the longitudinal test was not restricted to mature animals. Age-stratified comparison was not prespecified as a separate study endpoint. Because the four images obtained from each animal were repeated observations rather than independent biological samples, the effective group sizes were 32 adult cows and 16 calves. Variation in chronological age, birth date, and developmental stage further limited the interpretation of post hoc subgroup percentages. Reliable age-specific performance estimates will require larger, balanced cohorts with prospectively defined age categories.

Compared with recent cross-sectional benchmarks, the present sample was smaller, but the temporal protocol was more stringent. Shojaeipour et al. reported 99.11% identification accuracy in 300 cattle using a YOLOv3-ResNet50 pipeline [10]. Li et al. achieved 98.7% across 268 cattle and 4923 images, with 28.3 ms per image for the fastest model [11]. Those studies established the scalability of PN classification, whereas the present study adds a defined 131-day independent session. Multi-feature fusion can improve resilience when the muzzle or other traits are obscured [12], and open-set metric learning provides a more appropriate framework when unregistered cattle may enter the system [13]. These approaches address deployment questions that are complementary to the closed-set temporal question examined here.

Automated localization performed strongly for the muzzle class. The muzzle mAP@0.5 of 0.982, mean IoU of 0.96, and successful localization in all 232 detector validation and test images support the feasibility of standardized PN cropping. Straw was more difficult: recall was 0.578, and mean IoU was 0.76. This difference is biologically and visually plausible because the muzzle is a large, coherent anatomical region, whereas straw fragments are small, irregular, variably illuminated, and sometimes similar to reflected light or background structures. Straw-aware annotation may therefore assist quality control, but a straw detector should not be assumed to remove every contamination artifact.

A high-contrast stripe mask was explored during development using Otsu-based thresholding [18]. The reviewer correctly noted that such a texture could itself influence classifier attention. For this reason, comparative masking percentages and causal claims were removed from the revised manuscript. A rigorous occlusion study should compare solid fills, mean-color replacement, blur, inpainting, segmentation-based removal, random noise, and equal-area pseudo-masks, with paired image-level predictions and prespecified statistical analysis. Grad-CAM [18] may aid qualitative interpretation, but activation maps alone cannot demonstrate causal reliance on a particular texture.

The practical value of PN imaging lies in its non-invasive and intrinsic character. It does not require an implanted device and cannot be lost in the same manner as an external tag. However, the approach should be viewed as complementary rather than a replacement for legally established identifiers. A combined workflow could use an ear tag or radio-frequency identifier for registration and PN imaging for contact-free identification, n, discrepancy checking, recovery of identity after tag loss, or remote confirmation. Metric-learning approaches [29] may further allow new cattle to be enrolled without retraining a fixed 48-class softmax classifier.

The broader literature shows a progression from manual nose prints and veterinary descriptions [20,21,22,23,24,25,26,27,28,30], through texture descriptors, SIFT matching, graph methods, and hybrid classifiers [31,32,33,34,35], to deep convolutional and mobile recognition systems [36,37]. Recent work has expanded the field through crossbred-cattle evaluation, few-shot prototype learning, depth-based universal identification, and systematic analysis of livestock computer-vision datasets [38,39,40,41]. Across these approaches, four requirements remain decisive for practical adoption: independent temporal validation, explicit treatment of occlusion and image quality, rejection of unregistered animals, and transparent reporting of dataset composition.

This study has limitations. The cohort comprised 48 cattle from one university farm, with only four images per animal at each session. The 131-day interval supports short-term, not lifelong, persistence. The archived T1 result was pooled across adult cows and calves, so a separate age-group difference cannot be inferred. The experiment was designed as closed-set 1:N identification. Because the 48-class softmax model assigns each input to one of the enrolled identities and does not contain a calibrated rejection threshold, a valid open-set evaluation would require an independently sampled cohort of unenrolled cattle and a prespecified threshold-calibration procedure. The precise random seed used in the original classifier-development workflow could not be confirmed from the archived study records; this is acknowledged as a limitation for exact computational replication of the original training trajectory. Detector data were partitioned at image level, and external validation across farms, breeds, cameras, and operators remains necessary. Future studies should prospectively retain fixed random seeds, complete image-level split lists, repeated-seed evaluations, and open-set test cohorts. Finally, latency was measured on an NVIDIA L4 GPU and should not be generalized to mobile hardware without direct benchmarking.

## 5. Conclusions

A two-stage YOLOv12n-EfficientNetB0 framework successfully re-identified the same 48 cattle from PN images acquired in an independent session 131 days after baseline, correctly classifying 185 of 192 T1 images (96.35%; 95% CI: 92.67–98.22%). The result supports retention of identity-relevant PN characteristics over a short growth interval and demonstrates the feasibility of non-invasive PN-based identification in a cohort containing both adult cows and calves. Strong automated muzzle localization and low GPU inference latency further support operational potential. The method should presently be regarded as a complementary biometric-identification approach. Longer follow-up, age-stratified reporting, multi-farm external validation, open-set protocols, and testing on practical edge devices are required before routine use.

## Figures and Tables

**Table 1:** Comparison of recent cattle biometric studies relevant to muzzle-based identification.

Study	Scale	Temporal Interval	Independent Temporal Test	Occlusion/Robustness Strategy	Reported Inference
Shojaeipour et al., 2021 [10]	300 cattle; 2632 images	Not specified	No	Automated muzzle localization; mixed breeds	Not reported
Li et al., 2022 [11]	268 cattle; 4923 images	Not specified	No	Data augmentation and weighted loss	28.3 ms/image
Li et al., 2024 [12]	566 cattle; multimodal data	Not specified	No	Face–muzzle–ear-tag decision fusion for partial occlusion	Not reported
Wang et al., 2024 [13]	118 cows; 11,800 side-view images	Not specified	Open-set, not temporal PN testing	Spatial transformation and metric learning	Not reported
Present study	48 cattle; 384 longitudinal images; 775 detector images	131 days	Yes: all T1 images	Muzzle/straw detection and standardized cropping	8.3 ms/image

**Table 2:** Composition and role of the image datasets.

Dataset Component	Animals/Source	Images per Animal	Total Images	Role
Baseline (T0)	48 (32 adult cows, 16 calves)	4	192	Classifier development/enrollment: 134 training, 29 validation, and 29 internal-test images; all 48 identities represented in training
Follow-up (T1)	Same 48 cattle	4 new images	192	Independent 131-day temporal test
Detector: local	Independent slaughterhouse cattle	Variable	565	Muzzle/straw detector development
Detector: public	Zenodo dataset [14]	210 images from 30 identities	210	Muzzle/straw detector development

**Table 3:** Retained YOLOv12n detector-performance summary.

Outcome	Muzzle	Straw	Combined/Overall
Precision	0.890	0.734	0.812 (final epoch)
Recall	0.978	0.578	0.764 (final epoch)
mAP@0.5	0.982	0.588	0.785 best; 0.754 final
Mean IoU	0.96	0.76	-
Validation + test localization	232/232 muzzles localized	-	116 validation + 116 test

**Table 4:** Principal outcomes of the revised study.

Outcome	Result	Interpretation
Independent temporal re-identification	185/192 images; 96.35% (95% CI: 92.67–98.22%)	Same 48 cattle, 131 days after T0
Muzzle detection	Precision 0.890; recall 0.978; mAP@0.5 0.982	Strong automated PN localization
Muzzle localization review	232/232 validation and test images; mean IoU 0.96	Complete localization in detector evaluation subsets
End-to-end latency	8.3 ms/image	Measured on NVIDIA L4 GPU

## Data Availability

The 210 external images used for detector development are publicly available in the Muzzle Cow New Dataset on Zenodo (https://doi.org/10.5281/zenodo.7988559). The locally acquired raw images and an anonymized session-level image index are available from the corresponding author on reasonable request, subject to institutional requirements. The retained study archive includes the aggregate evaluation summaries reported in this article; complete image-level classifier prediction records and the original trained classifier model files are not available for sharing. This distinction clarifies the availability of the raw image data separately from the computational output files.
